# Hexagonal Close-Packed Au@Ag Superlattices for Versatile and Cost-Effective SERS Platforms

**DOI:** 10.3390/nano16060385

**Published:** 2026-03-23

**Authors:** Weizhe Fu, Yinan Zhang, Jiapeng Zheng

**Affiliations:** 1 School of Artificial Intelligence Science and Technology, University of Shanghai for Science and Technology, Shanghai 200093, China; 233350592@st.usst.edu.cn (W.F.); zhangyinan@usst.edu.cn (Y.Z.); 2 Institute of Photonic Chips, University of Shanghai for Science and Technology, Shanghai 200093, China

**Keywords:** Au@Ag nanospheres, plasmonic superlattices, surface-enhanced Raman scattering, interfacial self-assembly

## Abstract

The rapid fabrication of low-cost surface-enhanced Raman scattering (SERS) substrates is highly desirable for chemical and biological sensing. Existing customized SERS substrates, such as Au or Ag nanostructures produced by physical deposition, frequently involve complex fabrication routes, which limits the scalability of SERS devices. Here, we present the hexagonal close-packed plasmonic superlattices as an efficient, low-cost and applicable SERS platform, fabricated by scalable seed-mediated growth and interfacial self-assembly methods. We systematically compared Ag, Au, and Au@Ag nanospheres (NSs) of different sizes and demonstrated that the plasmonic superlattices made by 55 nm Au@Ag NSs exhibit the strongest Raman response, highest sensitivity, lowest detection limit, good spatial uniformity, and broad applicability. Simulations and Raman mapping experiments further confirm that Au@Ag NSs achieve an optimal balance between hotspot density and plasmonic field intensity, allowing for direct identification and quantification of diverse biochemical targets.

## 1. Introduction

Surface-enhanced Raman spectroscopy is a molecular analysis technique known for its ultra-high sensitivity and non-destructive detection capabilities, making it highly promising for chemical and biological applications [1,2,3]. Localized surface plasmon resonance (LSPR) in noble metal nanomaterials such as Au and Ag can enhance the electromagnetic field near the metal surface. The regions with highly confined electric field, termed “hotspots”, play a critical role in enhancing Raman signals of adsorbed molecules [4,5,6].

Although SERS technology has achieved a high level of maturity in laboratory research, its commercial and market applications still face challenges related to applicability, stability, and cost-effectiveness [7]. Currently, the mainstream commercial SERS substrates typically employ vapor deposition to fabricate Au or Ag nanostructures. Large-scale equipment is required, resulting in high costs that hinder mass production [8,9,10]. In contrast, plasmonic superlattices offer advantages such as operational flexibility, low cost, and ease of scaling up. These superlattices are generally made by the self-assembly of chemically synthesized NPs into close-packed nanostructures [11,12,13]. A variety of metal nanoparticles (NPs) have been successfully synthesized and have demonstrated excellent performance as the building blocks, including nanorods, nanostars, and nanobipyramids [14,15,16]. Other plasmonic NPs with complex morphologies such as bimetallic octapods, Au dual-rim nanoframes, and open cross-gap Au nanocubes were also employed [17,18,19]. These structures exhibit a high density of hotspots and excellent performance in SERS measurements; however, their complex preparation processes in morphology control still pose significant challenges for achieving cost-effective SERS analysis. Compared to complex nanostructures, spherical NPs are regarded as classical isotropic particles, and serve as the most fundamental and easily controllable model in both experimental and theoretical research [20,21].

Among various spherical building blocks, Au@Ag core–shell NSs have attracted widespread attention due to their low cost and excellent plasmonic performance. This structure can be readily synthesized via seed-mediated growth, which allows precise control over the thickness of Ag nanoshell [22]. In addition, Au@Ag NSs significantly reduce Au consumption, making them suitable for large-scale fabrication and practical applications. Developing a reliable, versatile, and cost-effective SERS substrate based on Au@Ag NS-superlattices would support the commercialization of this technology. Comprehensive research on the SERS performance of Au@Ag NS-superlattices is therefore required to verify the advantages of Au@Ag NS-superlattices [7].

In this work, we report a strategy for constructing hexagonal close-packed superlattices based on various plasmonic NSs. Ag, Au, and Au@Ag NSs of varying sizes were synthesized and assembled into superlattices via droplet evaporation-induced assembly (DEIA) methods. We systematically investigated their optical properties by Raman spectroscopy and finite difference time domain (FDTD) simulation. Theoretical and experimental results showed that Au@Ag NS-superlattices support the strongest LSPR effect. The hexagonal close-packed Au@Ag NS-superlattices enabled the SERS detection with the highest sensitivity and detection limit. Moreover, Au@Ag NSs were successfully assembled into uniform, large-area nanofilms via interfacial self-assembly. The resulting nanofilms show good chemical stability, as well as good uniformity and reproducibility in Raman sensing. Without requiring expensive vacuum deposition equipment, the hexagonal close-packed Au@Ag NS-superlattices present a highly promising SERS platform for trace chemical analysis, biosensing, and environmental monitoring.

## 2. Materials and Methods

### 2.1. Chemicals

Silver nitrate (AgNO_3_, 99.8%), hydrogen peroxide (H_2_O_2_, 30%), ammonia monohydrate (NH_4_OH, 25–28%), cyclohexane (C_6_H_12_, ≥99.7%), chloroauric acid (HAuCl_4_, ≥99%), and sodium borohydride (NaBH_4_, 99%) were purchased from Shanghai Chemical Reagent (Shanghai, China). Trisodium citrate (SC, ≥99%) and L-Ascorbic acid (AA, 99%), were purchased from Sigma-Aldrich (Shanghai, China). Hexadecyl trimethyl ammonium bromide (CTAB, 99%), rhodamine 6G (R6G, 95%), 4-Aminothiophenol (4-ATP, ≥98%), and tannic acid (TA) were purchased from Aladdin (Shanghai, China). Hexadecyl trimethyl ammonium chloride (CTAC, 99%) was purchased from Macklin (Shanghai, China). Ethanol (99%) was obtained from Adamas-beta (Shanghai, China). All chemicals were analytical grade and used as received without further purification. Deionized (DI) water (resistivity of 18.2 MΩ·cm) was used in all experiments.

### 2.2. Synthesis of Au NSs

Au nanoparticles were synthesized using the seed-mediated growth method adapted from the reported procedure [23]. Briefly, the seed solution was prepared by the rapid injection of freshly prepared NaBH_4_ (0.01 M, 0.60 mL) into the solution made by HAuCl_4_ (0.01 M, 0.25 mL) and CTAB (0.1 M, 9.75 mL) under vigorous stirring. The reaction was allowed to proceed under gentle stirring at room temperature for 3 h. The growth solution was then prepared by first mixing CTAB (0.1 M, 9.75 mL) and HAuCl_4_ (0.01 M, 4 mL) with DI water (190 mL). Ascorbic acid (AA, 0.1 M, 15 mL) was then added into the mixture, rapidly decolorizing the mixture under vigorous stirring. The seed solution was injected last and the reaction mixture was transferred to a 30 °C oven for 12 h. Au NPs with a diameter of 25 nm were therefore synthesized, then washed and concentrated. Subsequently, the Au NP solution was employed as seeds to yield NSs with relatively narrow size distributions, which were added to a growth solution containing CTAC (0.025 M, 10 mL), HAuCl_4_ (0.01 M, 0.5 mL), and ascorbic acid (AA, 0.1 M, 0.25 mL). CTAC was used to cause the isotropic growth of Au NSs. The mixture was stirred gently at 45 °C for 2 h. The obtained Au NSs were collected by centrifugation at 4500 rpm for 10 min and washed once with CTAB solution. The precipitate was then redispersed in CTAB solution (0.02 M, 35 mL) for storage. Different sizes of Au NSs were obtained by varying the amount of seed solution. Specifically, by decreasing the seed volume from 6 mL to 4.5, 3.5, and 3 mL, the final diameters of the Au NSs increased to approximately 45, 53, 63, and 71 nm, respectively.

### 2.3. Synthesis of Ag NSs

The Ag NSs were synthesized via a two-step method [24]. First, SC (0.1 M, 5 mL) and TA (0.01 M, 0.25 mL) were added into 94 mL of DI water under magnetic stirring. When the mixture temperature reaches 80 °C, AgNO_3_ (0.1 M, 0.75 mL) was injected, causing the solution to turn yellow immediately. The mixture was then heated with vigorous stirring in an 80 °C water bath for 15 min to obtain the Ag seed solution. Subsequently, 20 mL of this seed solution was extracted, diluted with 17 mL of DI water and heated to 90 °C. SC (0.1 M, 125 μL), TA (0.01 M, 400 μL), and AgNO_3_ (0.1 M, 260 μL) were sequentially injected at 1 min intervals. After 20 min of reaction, Ag NSs with a diameter of approximately 43 nm were obtained. This product was used as the seed solution for the next growth step. By repeating this process and adjusting the amounts of reagents and Ag precursor, Ag NSs with larger sizes, such as about 53, 60, and 71 nm, were successively synthesized. The obtained Ag NSs were collected by centrifugation at 6500 rpm for 10 min to remove the supernatant, and the precipitate was redispersed in deionized water for subsequent use.

### 2.4. Synthesis of Au@Ag NSs

Au@Ag core–shell nanospheres were synthesized by following the seed-mediated growth method [25]. Prepared Au NPs (25 nm diameter, optical density 2.2, 1 mL) and CTAC (0.08 M, 1 mL) were first mixed in a reaction vessel, and AgNO_3_ (0.01 M) and ascorbic acid (AA, 0.1 M) were then added in a 2:1 volume ratio. The mixture was stirred at 60 °C for 5 h, after which it was sonicated for 10 min and subsequently left undisturbed at 30 °C overnight. The resulting precipitate was then removed and the dispersion was centrifuged for 10 min. The collected particles were redispersed in CTAB solution (0.1 M, 40 mL). By keeping the amount of Au seeds constant, the thickness of the Ag shell could be tuned from 10 nm to 25 nm by varying the volumes of AgNO_3_ and AA. Specifically, AgNO_3_ volumes of 0.25, 0.5, 1.0, and 1.5 mL paired with AA volumes of 0.125, 0.25, 0.5, and 0.75 mL, respectively, yielded shells of approximately 10, 15, 20, and 26 nm in thickness.

### 2.5. Interfacial Self-Assembly

A total of 10 mL of the Au@Ag NSs solution was placed into the Petri dish, followed by careful layering of 5 mL of cyclohexane to form a biphasic system with an approximate thickness ratio of 1:2 (upper organic layer to lower aqueous nanoparticle layer). Ethanol was then injected to trigger the formation of Au@Ag NS-nanofilm at the interface. The interfacial nanofilm was collected onto ethanol-rinsed SiO_2_ substrates and allowed to dry for SERS measurement [26].

### 2.6. Etching of Ag Nanoshells

Silver etching was performed following a reported procedure [27]. Briefly, 5 mL of NH_4_OH was added to a Petri dish, and the SiO_2_ substrate coated with Au@Ag NSs was fully immersed in the solution. Then, 50 μL of H_2_O_2_ (10%) was gently introduced into the dish. After etching for 10, 20, or 30 min, the substrate was rinsed thoroughly, and prepared for further characterization.

### 2.7. Theoretical Simulation

Numerical simulations were carried out using the FDTD method. A plane wave source at 532 nm was employed to excite the Au NSs, Ag NSs, and Au@Ag NSs with different diameters. The permittivity and permeability values for Au and Ag were taken from the built-in material database. A frequency domain field monitor recorded the magnitude distribution of the electric field (|E_max_|/|E_0_|).

### 2.8. Characterization

UV–visible absorption spectra (300–600 nm) of plasmonic NSs were recorded at room temperature on a Shimadzu UV 2400 spectrophotometer (Kyoto, Japan). Morphological analysis was performed using a transmission electron microscope (JEOL 2100F, Kyoto, Japan) and scanning electron microscope (ZEISS Sigma 300, Jena, Germany). NP diameter and Ag-shell thicknesses were measured from SEM and TEM images using Nano Measurer software (Version 1.2). SERS measurements were conducted on a Horiba LabRAM Odyssey system equipped with a 532 nm laser. The laser power at the sample surface was 6 μW. A 50× objective lens was used for both excitation and signal collection, and the estimated laser spot area was approximately 0.33 μm^2^. For SERS testing, aqueous dispersions of NSs were deposited onto ethanol-cleaned SiO_2_ wafers and dried on a 40 °C hotplate to form the superlattices. Subsequently, R6G and 4-ATP solutions with concentrations ranging from 10^−4^ to 10^−9^ M were dripped onto the superlattices and dried prior to spectral acquisition. All spectra were collected with an integration time of 2 s and 1 accumulation per measurement. For SERS mapping measurements, the integration time and accumulation number were both set to 1 s and 1 accumulation per pixel, respectively. The mapping step size was set to 0.3 μm. All spectral data were processed using OriginPro software (Version 2024). A Savitzky–Golay smoothing filter (window size: 9 points; polynomial order: 2) was applied to reduce high-frequency noise. Baseline correction was performed using a fourth-order polynomial fitting method. The analytical enhancement factor (AEF) was calculated according to $AEF=(I_{SERS}/C_{SERS})/(I_{Raman}/C_{Raman})$, where $I_{SERS}$ and $C_{SERS}$ are the Raman intensity and concentration of R6G on the SERS substrate, respectively. $I_{Raman}$ was obtained from a R6G solution (0.01 M) on a glass substrate under identical measurement conditions [28].

## 3. Results and Discussion

### 3.1. SERS Platform Based on NS-Superlattices Fabricated by DEIA

**Figure 1:**
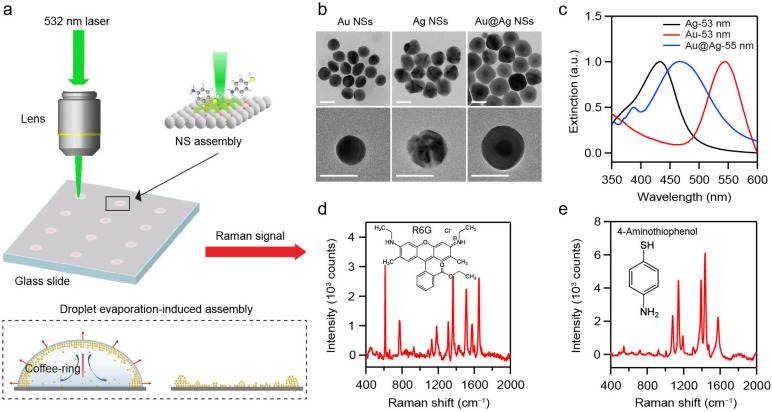
(**a**) Schematic illustration of SERS measurement based on plasmonic NS-superlattices. (**b**) TEM images of Au, Ag, and Au@Ag NSs. Scale bar: 50 nm. (**c**) Extinction spectra of Au, Ag, and Au@Ag NSs with diameter of about 55 nm in solution. (**d**,**e**) Raman spectra of R6G (**d**) and 4-ATP (**e**).

When assembled into densely packed superlattices, the monodisperse plasmonic NPs can form periodic architectures that yield excellent near-field enhancement within interparticle nanogaps. Uniformly distributed plasmonic hotspots can be generated, making plasmonic superlattices highly promising for SERS detection. Consequently, developing the versatile and cost-effective SERS platform relies on two key aspects: (1) a high-yield synthetic approach of plasmonic NPs and (2) a scalable assembly method of plasmonic NPs into ordered structures. To achieve scalable fabrication of SERS platforms, we synthesized various metal NSs using a seed-mediated growth method and assembled them into various superlattices based on self-assembly approaches (Figure 1a) [23,24,25]. Au, Ag, and Au@Ag NSs were synthesized as the building blocks in the study. Then, 25 nm Au NPs and 25 nm Ag NPs were first prepared as seeds for the synthesis of Au, Ag, and Au@Ag NSs. Au NSs were synthesized by reducing chloroauric acid with ascorbic acid [23]. Ag NSs were obtained based on the co-reduction of silver nitrate by sodium citrate and tannic acid [24]. Au@Ag NSs with core–shell nanostructures were fabricated by the overgrowth of Ag nanoshell onto the Au NPs [25]. Detailed synthesis procedures are provided in the Section 2. Transmission electron microscopy (TEM) and scanning electron microscopy (SEM) images (Figure 1b and Figure S1) demonstrate the high-yield synthesis of Au, Ag, and Au@Ag NSs with controllable diameter ranging from 40 to 80 nm. Extinction spectra (Figure S1) show that increasing the NS-diameter leads to a red shift in the LSPR band. Moreover, the plasmonic NSs of the same diameter but different compositions exhibit distinct LSPR wavelengths. As shown in Figure 1c, Ag, Au, and Au@Ag NSs with diameters of about 55 nm display the LSPR peaks at 430, 540, and 475 nm, respectively.

These plasmonic NSs were then assembled into three-dimensional superlattices via DEIA (Figure 1a). The coffee-ring effect and Marangoni effect can promote the spontaneous close-packing of the NSs [29,30]. Corresponding SERS spectra were acquired under the 532 nm laser excitation to evaluate the plasmonic performance of the resulting superlattices. Rhodamine 6G (R6G) and 4-Aminothiophenol (4-ATP) were employed as Raman probes. SERS measurements were performed on the superlattices with different configurations (Figure 1d,e). R6G was selected for its intense Raman signals and its widespread use as a benchmark in SERS studies [31]. 4-ATP was chosen as an additional probe to further evaluate the applicability of NS-superlattices [32]. All the collected Raman spectra were processed by smoothing with the Savitzky–Golay method and baseline-corrected by using a polynomial function to eliminate the background interference in the range of 400–2000 cm^−1^.

### 3.2. SERS Measurements on Superlattices Composed of Different NS Compositions

**Figure 2:**
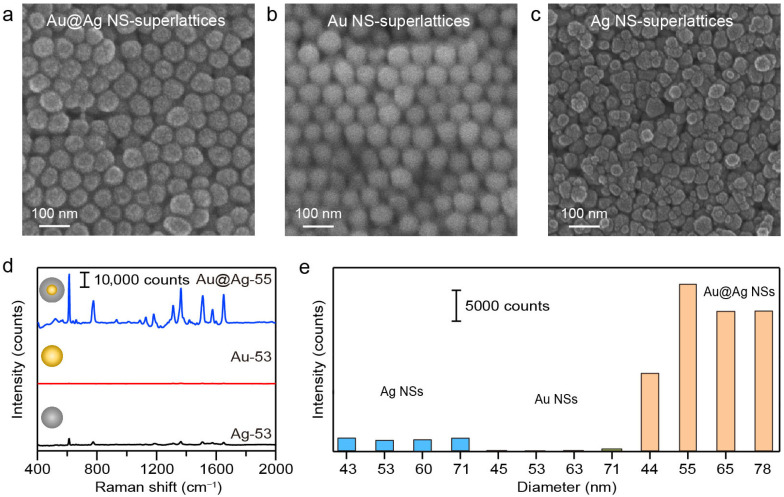
(**a**–**c**) SEM images of Au, Ag, and Au@Ag NS-superlattices fabricated by droplet evaporation-induced assembly. The diameters of Au, Ag, and Au@Ag NSs were 53.4 ± 4.9, 52.6 ± 6.1, 55.4 ± 3.6 nm, respectively. (**d**) Comparison of the SERS spectra of R6G molecules (100 nM) for Au, Ag, and Au@Ag NS-superlattices with NS-diameter of about 55 nm. (**e**) Raman intensities at the characteristic peak 613 cm^−1^ for the superlattices with different NS compositions and diameters.

We first conducted comparative measurements on plasmonic superlattices composed of different NS compositions to evaluate the influence of nanomaterials on SERS performance. The typical structures of Au, Ag, and Au@Ag NS-superlattices with varying NS-diameters were confirmed via SEM imaging. The structural order of superlattices depends strongly on the size and shape uniformity of the NS building blocks. Ag NSs exhibited a broader size distribution (Table S1) and rough surfaces (Figure 1b) compared with Au and Au@Ag NSs. Driven by entropy maximization and interparticle interactions, the hexagonal close-packed arrangements were commonly observed in the Au and Au@Ag NS-superlattices with NS-diameter of about 55 nm (Figure 2a,b). Ag NS-superlattices with a similar NS-diameter, on the other hand, exhibited a disordered assembly state (Figure 2c). Correlative SERS microscopy of R6G (100 nM) was subsequently employed to correlate the assembly morphology with plasmonic enhancement (Figure 2d, Figures S2 and S3). The Au@Ag NS-superlattice was found to yield the strongest SERS signal across all tested diameters ranging from 40 to 80 nm. As shown in Figure 2e, the SERS intensities at 613 cm^−1^ for Au@Ag NS-superlattices with NS-diameters of about 44, 55, 65, and 78 nm were 6.3, 16.1, 12.2, and 11 times higher than those of Ag NS-superlattices of corresponding sizes, and 59.3, 146.8, 133.1, and 61.8 times higher than those of Au NS-superlattices of corresponding sizes. The Raman intensities at the characteristic peak 613 cm^−1^ demonstrated the clear dependence of SERS activity on both the material composition and the spatial configuration of the superlattice [33].

**Figure 3:**
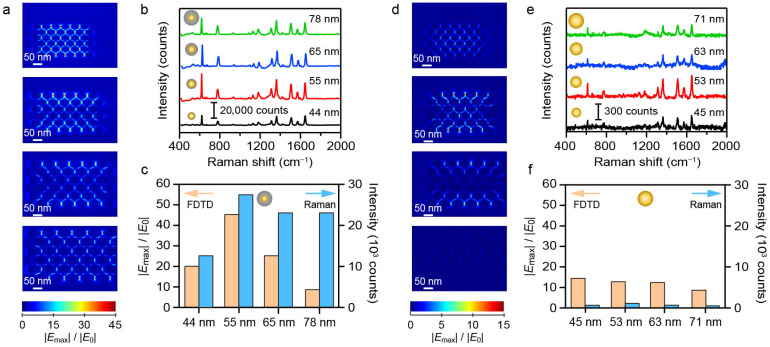
Comparison of the SERS performance between Au@Ag and Au NS-superlattices. (**a**–**c**) FDTD simulation and SERS measurement results of R6G molecules (100 nM) for the hexagonal close-packed Au@Ag NS-superlattices. (**d**–**f**) FDTD simulation and SERS measurement results of R6G molecules (100 nM) for the hexagonal close-packed Au NS-superlattices.

To investigate the effect of material composition on the SERS performance, we analyzed the Raman intensities and simulated electric field distributions of Au and Au@Ag NS-superlattices with different NS-diameters. As shown in Figure 3, the SERS intensity exhibited a distinct size dependence, initially increasing with diameter, peaking at 55 nm, and then decreasing for larger NSs. Notably, the 55 nm Au@Ag NS-superlattices yielded roughly twice the SERS intensity of the 45 nm counterparts (Figure 3b). Furthermore, the Au@Ag core–shell structure exhibited significantly stronger Raman signals than monometallic Au NSs. FDTD simulations were performed to calculate the localized enhanced electric fields. The models utilized geometric parameters that approximated the experimentally synthesized NSs, with a gap of 5 nm between adjacent NSs (Figure 3a,d). The simulated electric field distributions at the top surfaces of the NSs revealed the significant field enhancement within the interparticle nanogaps [34]. As shown in Figure 3c, the maximum electric field intensity for the 55 nm Au@Ag NSs was about twice that of the 45 nm structure. The simulations support the experimental observation that a thicker Ag nanoshell within a certain range leads to stronger SERS enhancement in the nanogaps of Au@Ag NSs. In comparison, the SERS performance of Au NSs showed relatively little variation, with a minor increase signal observed for the 53 nm NSs (Figure 3e,f). Overall, while all the tested NSs exhibited notable SERS properties, Au@Ag NSs consistently showed stronger enhancement than Au NSs across all size ranges.

**Figure 4:**
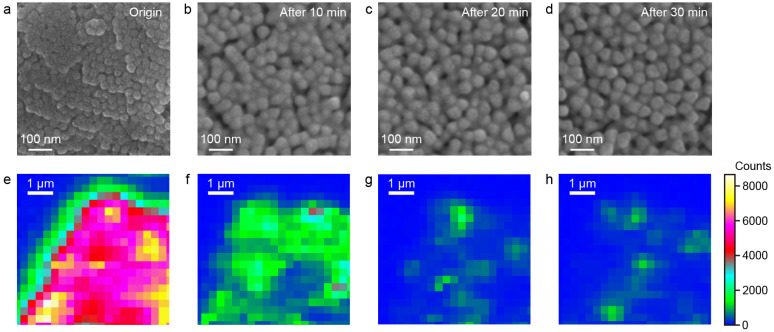
(**a**–**d**) SEM images of Au@Ag NS-superlattices etched at different times. (**e**–**h**) Raman mapping images of Au@Ag NS-superlattices etched at different times. The different scales reflect the distinct spatial resolutions of SEM and SERS mapping, which were used to characterize nanoparticle morphology and Raman signal distribution, respectively.

To investigate how structural variations influence the SERS performance, controlled Ag etching was performed on the Au@Ag NS-superlattices to modulate the surface morphology and local interparticle gaps within the assembled nanostructures. After self-assembling Au@Ag NS with a diameter of 55 nm on the silica substrate, the morphology change in superlattices was controlled through an etching process. Specifically, an etchant consisting of hydrogen peroxide and ammonium hydroxide was introduced to the substrate containing the Au@Ag NS-superlattices immersed in deionized water [27]. SEM images and SERS mapping were used to characterize the morphological evolution and changes in SERS performance of Au@Ag NS-superlattices over different etching durations. Ag atoms were preferentially removed from the outer nanoshells, leading to a reduction in the size of the Au@Ag NSs from 55 nm to 45 nm and a corresponding enlargement of the nanogaps (Figure 4a–d). Correspondingly, SERS mapping results (Figure 4e–h) show that the peak intensity at 613 cm^−1^ decreased with increasing etching time. It is noteworthy that the hexagonal close-packed arrangement of Au@Ag NSs was gradually replaced by a disordered structure during Ag-etching. Comparative spectra (Figure S4) indicate that after 30 min of etching, the Au@Ag NS-superlattices exhibited SERS signals of intensity equivalent to those of the Ag NS-superlattices, which can be attributed to their similar assembled morphology.

The experimental and FDTD simulation results in Figure 3 and Figure 4 demonstrated that the observed SERS signal intensity was predominantly governed by the electromagnetic field enhancement originating from the superlattices. The LSPR effect of metal NSs and interparticle nanogaps play a pivotal role in maximizing electromagnetic field enhancement and optimizing overall SERS intensity. On the one hand, Ag exhibits stronger field enhancement than Au, causing the amplification of the SERS signal in Ag-containing nanostructures [35,36]. On the other hand, a hexagonal close-packed configuration can generate the highest density of hotspots [26], leading to stronger SERS signals of Au@Ag NS-superlattices compared with Ag NS-superlattices. Additionally, the interparticle nanogaps can be modulated by varying the NS sizes. An increase in NS sizes reduces the number of NSs that can be accommodated in a fixed illumination area. The larger NSs provide fewer hotspots per unit area, resulting in a weaker SERS signal, whereas smaller NSs generally exhibit a lower electromagnetic field enhancement factor, which likewise diminishes SERS signals. The interplay between LSPR effect of Ag nanoshells and the hexagonal close-packed structure establishes an optimal condition for the strongest SERS signal. Therefore, 55 nm Au@Ag NS-superlattices exhibited Raman signal enhancements, approximately 11-fold and 90-fold greater than those of similarly sized Ag NSs and Au NSs, respectively.

### 3.3. Detection Limit and Sensitivity of SERS Signal

**Figure 5:**
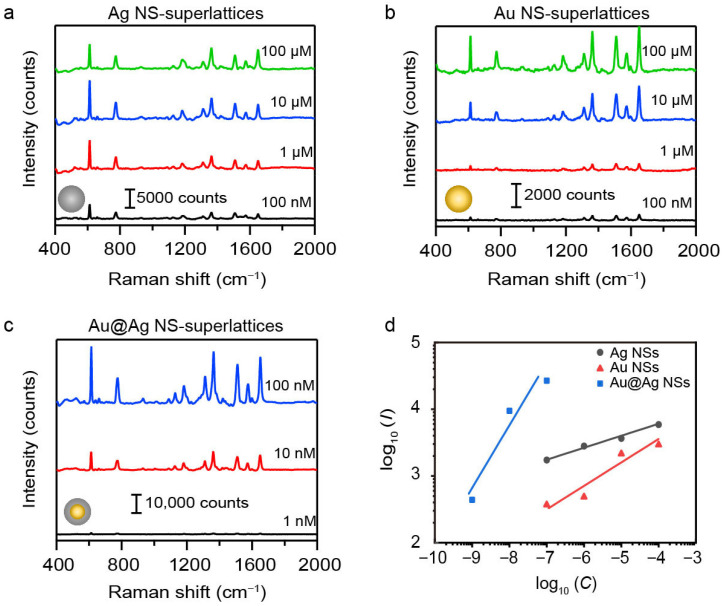
(**a**–**c**) SERS spectra of R6G molecules in different plasmonic metastructures, including Ag NS (**a**), Au NS (**b**), and Au@Ag (**c**) NS-superlattices. All the NSs have the same diameters of about 55 nm. (**d**) The linear relationship between the Raman intensity (*I*, in counts) and the concentration (*C*, in mol·L^−1^) of R6G on a logarithmic (log10) scale.

The detection limit and sensitivity of SERS signal were also systematically evaluated to further demonstrate the superiority of the 55 nm Au@Ag NS-superlattices. We collected the SERS spectra of R6G at concentrations ranging from 10^−9^ M to 10^−4^ M using NS-superlattices with different compositions and diameters (Figures S5–S7). Figure 5a–c present the raw SERS spectra for Au, Ag, and Au@Ag NS-superlattices with NS-diameter of about 55 nm. As the R6G concentration decreased from 10^−4^ M to 10^−9^ M, the intensities of characteristic Raman peaks gradually diminish. The limit of detection (LOD) for R6G was calculated based on the signal-to-noise (S/N) criterion (S/N ≥ 3). For the Au and Ag NS-superlattice substrates, the S/N of the characteristic peaks at 613 cm^−1^ exceeded 3 at 10^−7^ M, but fell below 3 at 10^−8^ M, indicating an LOD of 10^−7^ M. In contrast, the Au@Ag NS-superlattice substrate maintained a S/N larger than 3, even at 10^−9^ M, indicating an LOD of at least 10^−9^ M (detailed S/N values are provided in Table S2). The analytical enhancement factor (AEF) was estimated based on the measured signal intensities and the concentration of R6G molecules. The Au@Ag NS-superlattices exhibited an AEF of approximately 10^7^ at 10^−9^ M, whereas the Au and Ag NS-superlattices showed AEFs of 10^4^ and 10^5^, respectively, at 10^−7^ M (detailed AEF values are shown in Table S3). This demonstrates the superior detection capability of the Au@Ag core–shell structure compared with single-metal NSs. The sensitivity of SERS detection was examined by plotting the intensity of Raman peaks at 613 cm^−1^ against R6G concentration on a logarithmic (log_10_) scale. Figure 5d shows the linear relationship between the logarithmic SERS intensity (log_10_I) and the logarithmic concentration of R6G (log_10_C) for different superlattices with NS-diameter of about 55 nm. The Au@Ag NS-superlattices exhibit the steepest slope among the three samples (*k*_Au@Ag_ ≈ 0.89, *k*_Ag NSs_ ≈ 0.17, and *k*_Au NSs_ ≈ 0.33), indicating the highest detection sensitivity.

The superlattices assembled by 55 nm Au@Ag NSs thus combine the strongest Raman response, the optimal sensitivity, and the lowest detection limit. This performance is attributed to a synergistic effect between the LSPR effect of Ag nanoshells and hexagonal close-packed structure, as demonstrated before (Figure 2, Figure 3 and Figure 4). Similar experimental results can also be obtained when the 4-ATP was used as the SERS probe (Figures S8 and S9), showing the broad applicability of Au@Ag NS-superlattices in SERS-based biosensing.

### 3.4. SERS Measurement on NS-Superlattices Fabricated by Interfacial Self-Assembly

**Figure 6:**
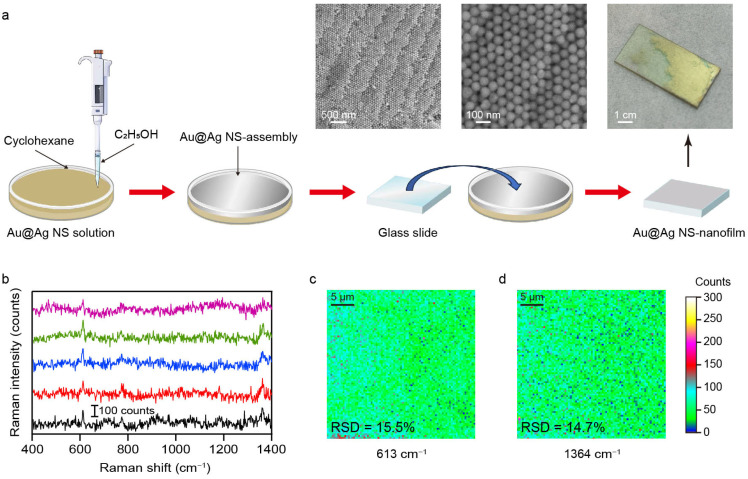
(**a**) Schematic diagram of interface self-assembly. The physical image and SEM images show the Au@Ag NS-nanofilm on the silica substrate. (**b**) Raw SERS spectra of R6G (100 nM) on the 5 random illuminated areas of Au@Ag NS-nanofilm. The multicolored plots indicated the different collected regions. (**c**,**d**) Raman mapping images for the characteristic peaks at 613 cm^−1^ and 1364 cm^−1^. The relative standard deviations (RSDs) are 15.5% and 14.7%, respectively.

While plasmonic superlattices fabricated by droplet evaporation-induced assembly enable high-sensitivity analyte detection, their use in large-scale quantitative SERS analysis remains limited by the large deviation in Raman signal intensity (Figure 4). The interfacial self-assembly method offers a versatile and practical route for constructing SERS-active superlattices [37,38]. This technique utilizes a liquid–air or liquid–liquid interface as a dynamic, a soft template to direct nanoparticles into large-area, and hexagonal close-packed nanofilms. Au@Ag NSs were therefore utilized as functional building blocks in the subsequent interfacial self-assembly process. As illustrated in Figure 6a, the introduction of a small amount of ethanol into a preformed cyclohexane/water bilayer system promptly triggered the assembly of Au@Ag NSs at the liquid–liquid interface. Driven by interfacial tension gradients, the Au@Ag NSs spontaneously reorganized into a densely packed, continuous nanofilm at the boundary [39]. A pre-cleaned glass substrate was then tilted and slowly immersed beneath the interfacial layer, followed by careful withdrawal to transfer the Au@Ag NS-nanofilm onto the solid substrate. This self-assembly approach ensures both macroscopic uniformity and structural integrity of the Au@Ag NS-nanofilm, as confirmed by the optical and SEM images shown in Figure 6a. The nanofilm generates a high density of uniformly distributed plasmonic hotspots, which ensures highly sensitive and reproducible SERS signals. The spatial uniformity of the self-assembled Au@Ag NS-nanofilm was further evaluated by Raman mapping analysis. As shown in Figure 6b–d, the Raman intensity maps for the characteristic peaks at 613 cm^−1^ and 1364 cm^−1^ show remarkable spatial uniformity across a 25 μm × 25 μm scan area. To quantitatively assess signal uniformity, 145 spectra were randomly collected within the mapped region, and the relative standard deviation (RSD) values of the Raman intensities were calculated (Figure S10). The RSD values were 15.5% at 613 cm^−1^ and 14.7% at 1364 cm^−1^, respectively, indicating good signal reproducibility and repeatability. The minimal intensity variation across this region confirms the excellent homogeneity of the SERS substrate, which is essential for reliable and reproducible molecular detection. These findings collectively demonstrate that the self-assembled Au@Ag NS-nanofilm constitutes a uniform plasmonic surface capable of delivering stable and consistent SERS enhancement across macroscopic dimensions. Moreover, the long-term chemical stability of the substrate was examined by comparing the SERS spectra measured at the initial stage and after six months of storage. The Raman signal retained about 50% of its performance, demonstrating that the substrate maintains stable SERS activity over time (Figure S11). The future integration of Au@Ag NS-nanofilm into a portable Raman spectrometer could hold great promise for on-site sensing in fields such as environmental surveillance, biochemical diagnostics, and industrial quality control [40].

## 4. Conclusions

In conclusion, this study has identified practical and cost-effective SERS-active superlattices through a systematic comparison of Ag, Au, and Au@Ag NSs across various dimensions. Both experimental measurements and FDTD simulations reveal that hexagonal close-packed Au@Ag NS-superlattices achieve an optimal balance between hotspot density and plasmonic field intensity. Specifically, Au@Ag NS-superlattices with NS-diameter of 55 nm exhibit the strongest Raman response, highest sensitivity, lowest detection limit, and broad applicability. Importantly, the Au@Ag NSs were successfully assembled into uniform and large-area monolayer films via the interface self-assembly method, enabling substrate fabrication without expensive vacuum deposition equipment. By integrating high sensitivity, chemical stability, good spatial uniformity, and economic viability, Au@Ag-based superlattices represent a highly promising SERS platform for practical applications such as trace chemical analysis, biosensing, and environmental monitoring.

## Data Availability

The original contributions presented in this study are included in the article/Supplementary Materials. Further inquiries can be directed to the corresponding author.

## Supplementary Materials

The following supporting information can be downloaded at https://www.mdpi.com/article/10.3390/nano16060385/s1.
