# Supplementary material for: Hexagonal Close-Packed Au@Ag Superlattices for Versatile and Cost-Effective SERS Platforms
Source: Nanomaterials (Basel). 2026 Mar 23;16(6):385. doi: 10.3390/nano16060385 (PMC13029661; doi:10.3390/nano16060385)
Supplement: Supplementary file 1 [file nanomaterials-16-00385-s001.zip › nanomaterials-4183670-supplementary.pdf]

Supplementary Information

Hexagonal close-packed Au@Ag superlattices for versatile and cost-effective SERS platforms

Weizhe Fu[1,2], Yinan Zhang[1,2], Jiapeng Zheng[1,2,*]

[1]School of Artificial Intelligence Science and Technology, University of Shanghai for Science and Technology, Shanghai 200093, China

[2]Institute of Photonic Chips, University of Shanghai for Science and Technology, Shanghai 200093, China

*Correspondence: jpzheng@usst.edu.cn (J.Z.)

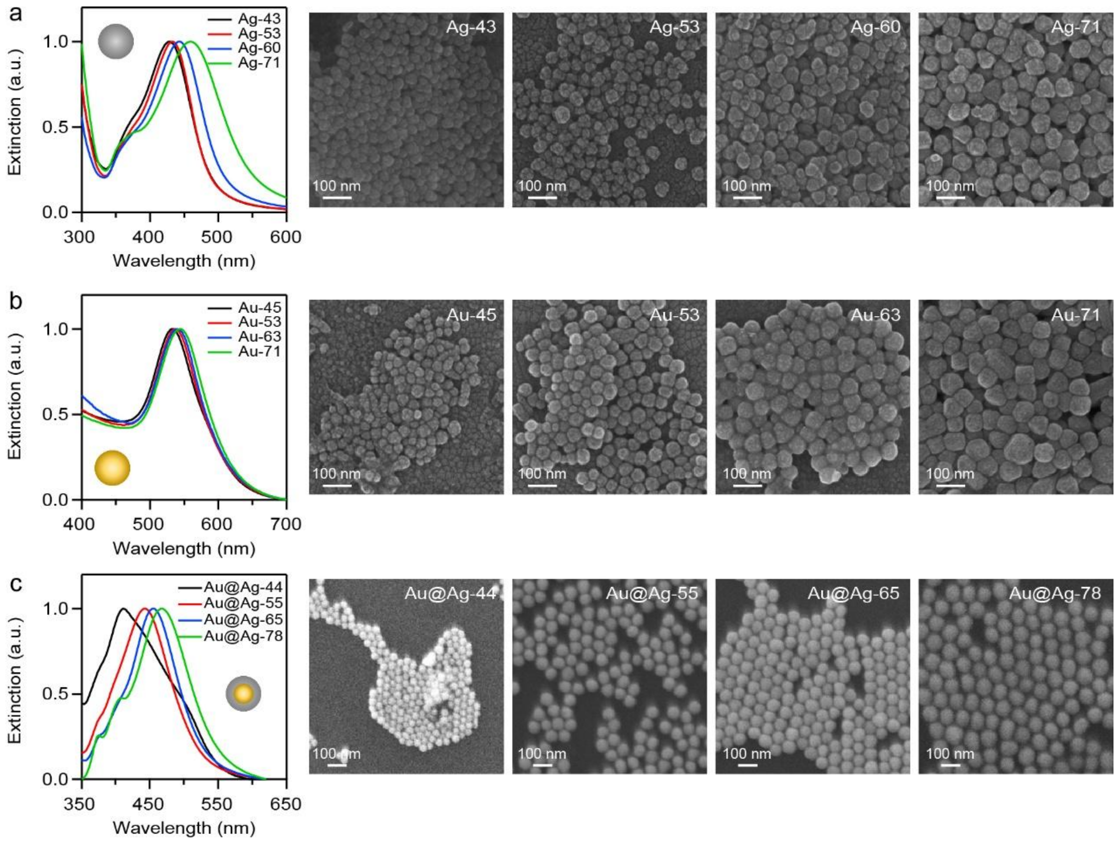

Figure S1. Extinction spectra and SEM images of Ag (a), Au (b), and Au@Ag (c) NSs with different diameters.

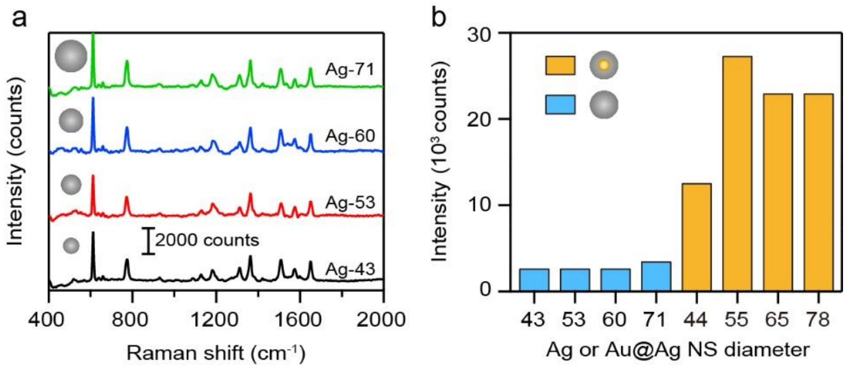

Figure S2. (a) SERS spectra of R6G molecule (100 nM) on Ag NS-superlattices with different NS-diameters. (b) Raman intensities at the characteristic peak 613 cm^{-1} for Ag and Au@Ag NS-superlattices with different diameters.

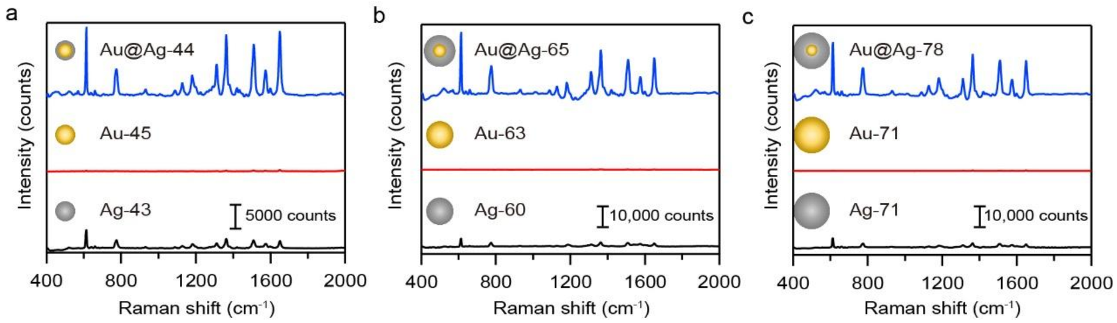

Figure S3. SERS spectra of R6G molecules (100 nM) on Ag, Au, and Au@Ag NS-superlattices with different NS-diameters.

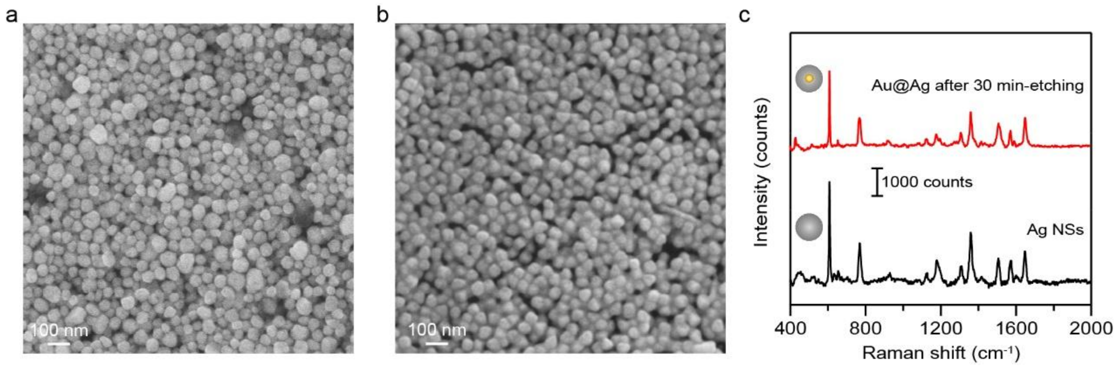

Figure S4. (a, b) SEM images of Ag NS-superlattices (a) and Au@Ag NS-superlattices after 30-min etching (b). (c) SERS spectra of R6G molecules on Ag and Au@Ag NS-superlattices after 30-min etching.

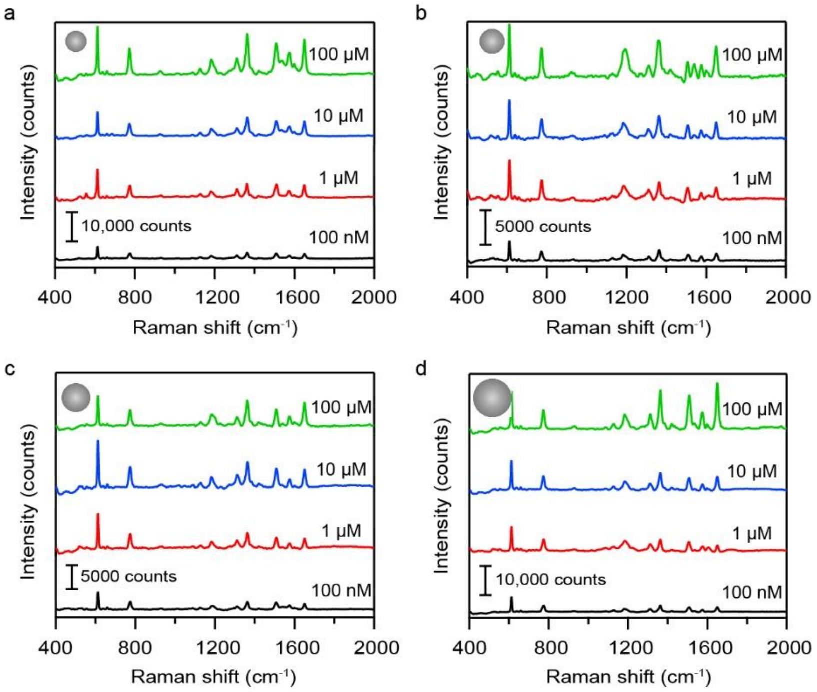

Figure S5. SERS spectra of R6G molecules with different concentrations on Ag NS-superlattices with NS-diameters of 43 (a), 53 (b), 60 (c), and 71 nm (d).

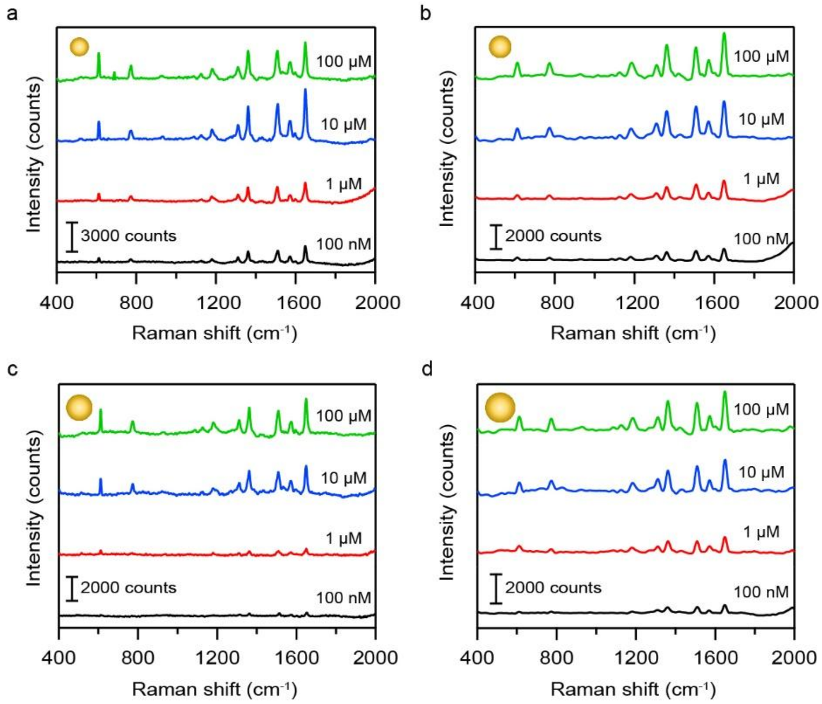

Figure S6. SERS spectra of R6G molecules with different concentrations on Au NS-superlattices with NS-diameters of 45 (a), 53 (b), 63 (c), and 71 nm (d).

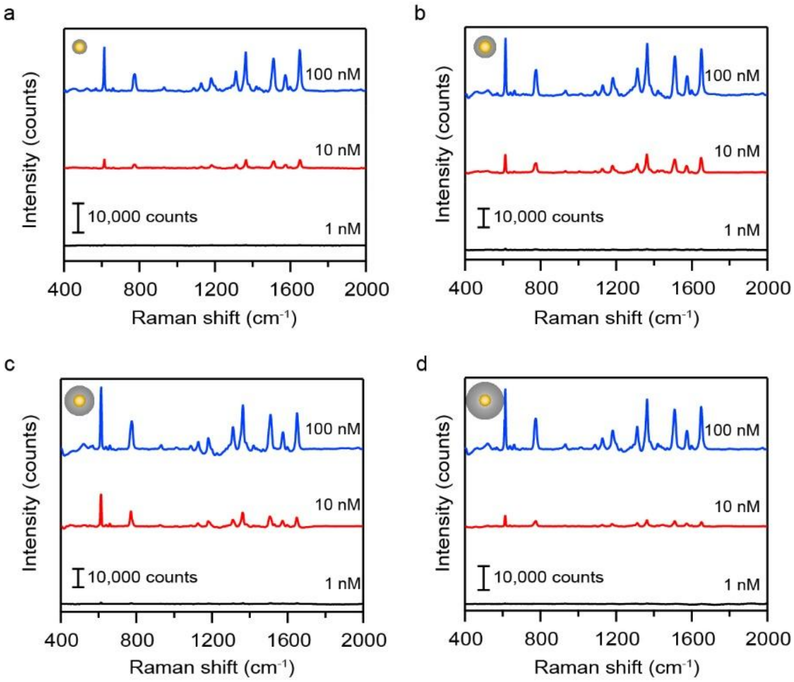

Figure S7. SERS spectra of R6G molecules with different concentrations on Au@Ag NS-superlattices with NS-diameters of 44 (a), 55 (b), 65 (c), and 78 nm (d).

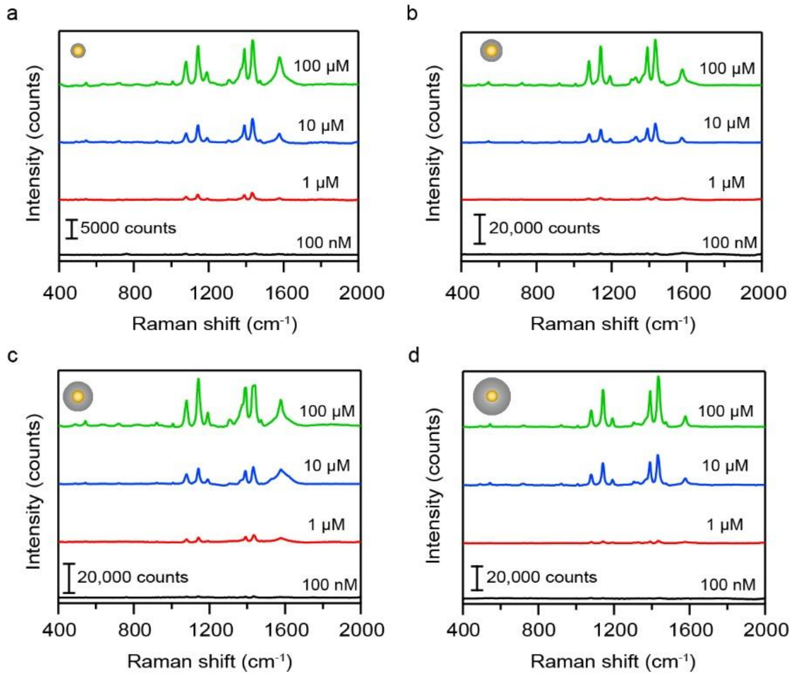

Figure S8. SERS spectra of 4-ATP molecules with different concentrations on Au@Ag NS-superlattices with NS-diameters of 44 (a), 55 (b), 65 (c), and 78 nm (d).

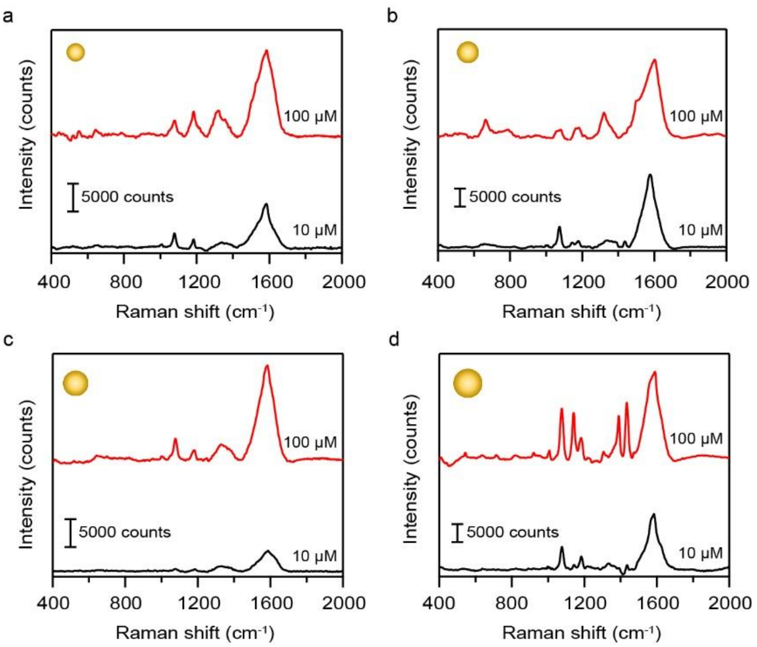

Figure S9. SERS spectra of 4-ATP molecules with different concentrations on Au NS-superlattices with NS-diameters of 45 (a), 53 (b), 63 (c), and 71 nm (d).

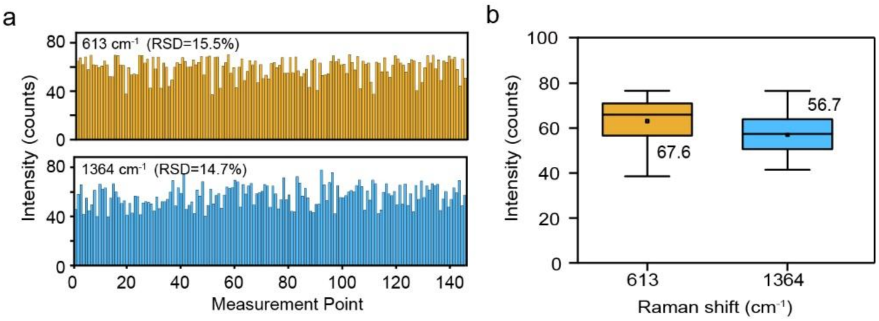

Figure S10. Relative standard deviation (RSD) of the SERS intensities. (a) Raman intensities collected from 145 randomly selected measurement points for the characteristic peaks at 1364 cm^{-1} and 613 cm^{-1}. (b) RSD of the SERS intensities.

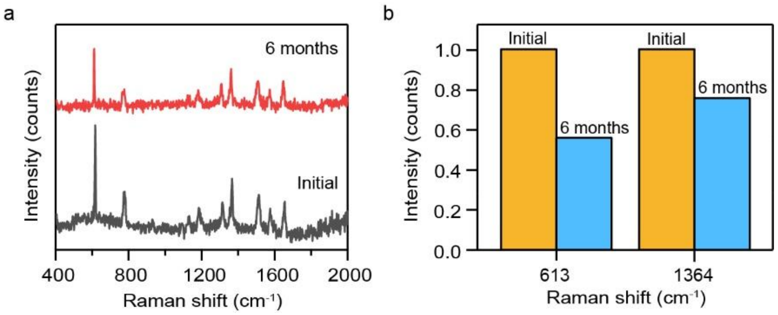

Figure S11. (a) SERS spectra of Au@Ag NS-superlattices measured at the initial stage and 6 months. (b) The normalized Raman intensities at 613 and 1364 cm^{-1}.

NS	Size distribution (nm)
Ag-43	43.0±5.5
Ag-53	52.6±6.1
Ag-60	60.0±6.6
Ag-71	71.0±6.9
Au-45	44.6±4.8
Au-53	53.4±4.9
Au-63	63.3±5.2
Au-71	70.8±7.5
Au@Ag-44	44.1±3.0
Au@Ag-55	55.4±3.6
Au@Ag-65	65.3±3.9
Au@Ag-78	78.0±4.8

Table S1. Size distribution of Au, Ag, and Au@Ag NSs shown in Fig. S1.

Concentration (M)	S/N (Au NSs)	S/N (Ag NSs)	S/N (Au@Ag NSs)
10^{-7}	14.62	68.35	-
10^{-8}	1.06	1.50	131.75
10^{-9}	-	-	26.36

Table S2. Signal-to-noise ratio (S/N) and limit of detection (LOD) determination. (“-” indicates undetectable signal or beyond the measurable range)

Substrate (SiO_2) with	R6G concentration (M)	Raman intensity at 613 cm^{-1} (counts)	AEF
Au NSs	10^{-7}	268.34	3.20×10^{4}
Ag NSs	10^{-7}	3265.11	3.89×10^{5}
Au@Ag NSs	10^{-9}	986.40	1.17×10^{7}
None	10^{-2}	839.80	N/A

Table S3. Estimated SERS analytical enhancement factors (AEF) for Au, Ag, and Au@Ag NS-Superlattices.
